# Results from 28 years of newborn screening for congenital adrenal hyperplasia in Sapporo City

**DOI:** 10.1186/1687-9856-2013-S1-P115

**Published:** 2013-10-03

**Authors:** Katsura Ishizu, Akie Nakamura, Kaori Fujikura, Masaru Fukushi, Tomoyuki Hotsubo, Taiko Sasaki, Toshihiro Tajima

**Affiliations:** 1 Department of Pediatrics, Hokkaido University School of Medicine, Sapporo, Japan; 2 Sapporo City Institute of Public Health, Sapporo, Japan; 3 Sapporo Immunodiagnostic Laboratory, Sapporo, Japan; 4 Department of Pediatrics, NTT East Japan Sapporo Hospital, Sapporo, Japan

## 

Newborn mass screening (MS) for congenital adrenal hyperplasia (CAH) was started in 1989 at nationwide of Japan. To date, the goals for prevention of life-threatening salt-wasting crises and prevention of incorrect sex assignment in virilized females are almost achieved. However in Sapporo city, which is located in the northern part of Japan, a pilot study was started in 1982 after the assessment of its feasibility and efficiency.

Here we report the results of MS from 1982 to 2010 in Sapporo city.

In 28 years, 17-hydroxyprogesterone (17-OHP) was determined in MS samples in 498,147 newborns and a total of 2,466 screened newborns had abnormal 17-OHP. Among them, 26 patients were diagnosed with 21-hydroxylase deficiency (21-OHD), which corresponds to a prevalence of 1:19,160 live birth in Sapporo city. It is almost identical to the worldwide incidence of CAH.

Among 26 patients, 20 patients were classified into salt-wasting forms, 5 patients were classified into simple virilizing forms and one was classified into nonclassic forms. 19 patients were female and 7 patients were male. Two familial cases were detected. The mean examination day of 25 patients (data of one patient was not available) was 6.4 days after birth (from 0 to 18 days). Among 18 female patients, 11 patients were clinically suspected because of virilization prior to MS result becoming available and were referred to neonatal center. 4 female patients were referred after MS screening. One female patient was assigned to male after birth; however the patient was diagnosed as having 21-OHD by MS and the sex was changed after the diagnosis. One female patient was a familial case and during pregnancy prenatal treatment was done and her genitalia was normal female. Another one patient having normal female genitalia and no symptom, was diagnosed as nonclassic forms by MS. By contrast to female patients, all 7 male patients were detected by MS.

Another type of CAH was not detected in our study.

In conclusion, MS for CAH in Sapporo city can be considered reliable.

